# The role of Snell’s law for a magnonic majority gate

**DOI:** 10.1038/s41598-017-08114-7

**Published:** 2017-08-11

**Authors:** Naoki Kanazawa, Taichi Goto, Koji Sekiguchi, Alexander B. Granovsky, Caroline A. Ross, Hiroyuki Takagi, Yuichi Nakamura, Hironaga Uchida, Mitsuteru Inoue

**Affiliations:** 1 Department of Electrical and Electronic Information Engineering, Toyohashi University of Technology, 1-1 Hibari-Ga-Oka, Tempaku, Toyohashi, Aichi 441-8580 Japan; 2 JST, PRESTO, 4-1-8 Honcho, Kawaguchi, Saitama, 332-0012 Japan; 3 Department of Physics, Keio University, Yokohama, 223-8522 Japan; 4 Faculty of Physics, Moscow State University, Leninskie Gory, Moscow, 119992 Russia; 5 Department of Materials Science and Engineering, Massachusetts Institute of Technology, 77 Massachusetts Avenue, Cambridge Massachusetts, 02139 USA

## Abstract

In the fifty years since the postulation of Moore’s Law, the increasing energy consumption in silicon electronics has motivated research into emerging devices. An attractive research direction is processing information via the phase of spin waves within magnonic-logic circuits, which function without charge transport and the accompanying heat generation. The functional completeness of magnonic logic circuits based on the majority function was recently proved. However, the performance of such logic circuits was rather poor due to the difficulty of controlling spin waves in the input junction of the waveguides. Here, we show how Snell’s law describes the propagation of spin waves in the junction of a Ψ-shaped magnonic majority gate composed of yttrium iron garnet with a partially metallized surface. Based on the analysis, we propose a magnonic counterpart of a core-cladding waveguide to control the wave propagation in the junction. This study has therefore experimentally demonstrated a fundamental building block of a magnonic logic circuit.

## Introduction

Magnonics is an emerging research field dealing with transmitting, processing, and storing information in ferromagnets in which spin waves propagate^1, 2^. Because the flow of spin waves can occur independently of the electron transport, even magnetic insulators, e.g. yttrium iron garnet (YIG), are capable of transmitting spin waves^3–5^. The possibility of reducing the Joule loss and accompanying heat generation has attracted attention owing to the possibility of realizing low-power computing^6^. Formalism and tunability of magnonic bands facilitate flexible control of signal transmission in spin wave devices^7^. In the early telecommunication market, filters, delay lines, and similar microwave components based on spin waves have been explored^8^. Rapid advances of nanotechnology in recent years have contributed to the control of spin waves in microstructures^9–11^, and the positive utilization of phase information motivated the development of logic elements^12, 13^.

In contrast to optical logic circuits, the on-chip compatibility of magnonic devices with electronic circuits holds great promise for encoding information into spin waves. In addition, such wave-based computing enables a flexible circuit design, for applications such as cellular nonlinear networks^14^, magnonic holography^15^, and multivalued logic^16^. In those applications, the control of superposition of waves is important, and thus, spin wave interferometers have been intensively investigated for possible use in computing^17–21^. Recently, the functional completeness of magnonic logic was demonstrated using three-input majority gates^22–25^. In fact, commercial computers based on majority gates have been fabricated^26^. Accordingly, a magnonic majority gate is one of the key elements to process phase information of spin waves in Boolean algebra. Such magnonic majority gates are usually realized with a combination of multiple input waveguides, where spin waves enter the input junction from different angles and are steered to the output waveguide. However, the behaviour of spin waves in the junction has not been clearly explained, and the incident angle requirements limit possible network geometries.

To control spin wave propagation in the junction, isotropy of dispersion curves in the device plane is crucial. Forward volume spin waves satisfy the requirement of isotropy, but backscattered waves from the end of the waveguide created a challenge. In a previous report, such backscattered waves were suppressed by using thin-gold attenuators, and two-wave interferometry of forward volume spin waves was realized with a linear waveguide^27^. This enabled experimental study of three-wave interferometry in a Ψ-shaped waveguide. Using this technique, we have experimentally demonstrated a magnonic majority gate based on the interferometry of forward volume spin waves. Because of the isotropy of the forward volume spin waves, their propagation in the input junction is reasonably explained by Snell’s law. The magnonic counterpart of a core-cladding waveguide is proposed to control the diffraction of spin waves within the junction. These results pave the way to build device networks composed of spin wave interferometers.

## Results

**Figure 1:**
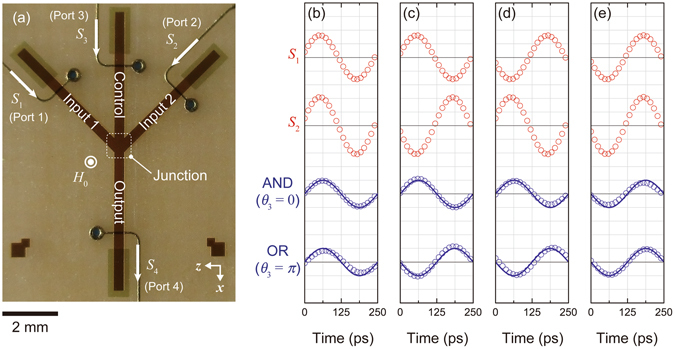
Magnonic majority gate realized by Ψ-shaped spin wave interferometer. (**a**) Overview of fabricated Ψ-shaped interferometer. Waveguide ends are covered by 10-nm-thick gold. Signals *S*_1_, *S*_2_, and *S*_3_ represent input waves, and *S*_4_ represents the output wave. All signals can be written in the sinusoidal form as *S*_*i*_ = *A*_*i*_ sin(*ωt* + *θ*_*i*_), and are given by microstrip lines with short termination. Input and output waveforms with different injection phases are also displayed: (**b**) (*θ*_1_, *θ*_2_) = (0, 0), (**c**) (0, π), (**d**) (π, 0), and (**e**) (π, π). Red circles represent input waveforms *S*_1_ and *S*_2_, blue circles represent output waveform *S*_4_, and bold lines represent theoretical curves calculated with *A*_*i*_ = 10 mV peak to peak. Vertical grid corresponds 50 mV/div. for red circles, and 5 mV/div. for blue circles except for *θ*_3_ = 0 in (b) and *θ*_3_ = π in (**e**). These waveforms are visualized in 15 mV/div.

Figure 1(a) shows the appearance of the fabricated Ψ-shaped interferometer. The interferometer consisted of 10-μm-thick monocrystalline YIG film grown on a gadolinium gallium garnet substrate. The Ψ-shaped waveguide was composed of 3 input ports, namely input 1, input 2, and control, along with an output port. Both input 1 and input 2 entered the junction area with 45° angle of incidence. This ridge waveguide was fabricated using photolithography and a micro-sandblasting technique. The waveguide was magnetized perpendicular to the plane so that forward volume spin waves were excited. The designed waveguide width of 350 μm was only capable of primary mode excitation at the operating frequency of 4 GHz. (At this frequency, the forward volume spin waves in the YIG are dipolar mode.) In addition, the fluctuation of the operation frequency and dispersion curve due to the measurement environment including thermal drift was suppressed in order not to excite higher modes. Thus any disturbance of the waves caused by the contribution of higher modes was prevented^28^. Injected spin waves were attenuated at the ends of the waveguide by covering the YIG surface with a 10-nm-thick gold layer formed by magnetron sputtering and lift-off processes. These attenuators reduced the amplitude of backscattered spin waves to less than 10% (See Methods). The Ψ-shaped waveguide was mounted by flip-chip bonding to a transducer composed of 50-μm-wide microstrip lines with short terminations. The distance from each microstrip line to the centre of the junction area was *L* = 3 mm. The incident angle of 45° yielded sufficient clearance between input microstrip lines to suppress the electromagnetic crosstalk (direct coupling among the microstrip lines) to below −40 dB. These microstrip lines excite spin waves with a broad range of wavelengths which are selected by the operating frequency of 4 GHz.

The output phase *θ*_4_ can be derived by linear superposition of sinusoidal input waves with phase *θ*_*i*_, including the phase rotation during propagation of waves:

1 $${\theta }_{4}={\tan }^{-1}\{\sum _{i=1}^{3}\sin ({\theta }_{i}-kL)/\sum _{i=1}^{3}\cos ({\theta }_{i}-kL)\}-kL+{\phi }_{0},$$

where *k* is the wavenumber of the spin waves, and *ϕ*_0_ is the phase offset in the external microwave circuit. According to Eq. (1), the deviation of output phase was given by $d{\theta }_{4}/dk=-2L$, and the stability of *k* was crucial in the experiment. Therefore, drifts of the bias magnetic field and the waveguide temperature were suppressed within 3095 ± 0.2 Oe and 27 ± 0.03 °C, respectively. When input phases *θ*_i_ are given as the binary values of 0 or π, the output phase *θ*_4_ represents the majority of *θ*_i_: MAJ(*θ*_3_,*θ*_2_,*θ*_1_). Based on majority logic, the logical product can be realized by MAJ(0, *θ*_2_, *θ*_1_) = AND(*θ*_2_,*θ*_1_), and the logical sum can be realized by MAJ(π,*θ*_2_,*θ*_1_) = OR(*θ*_2_, *θ*_1_), where *θ*_3_ is used as a control input.

Figure 1(b–e) show the results of three wave interferometry for AND operation and OR operation. The amplitudes for each of the sine curves are shown with different vertical axis scales. In the experiment, the phase offset *ϕ*_0_ was adjusted to compensate the phase rotation caused by spin wave propagation. The output phase was *θ*_4_ = π only when (*θ*_1_, *θ*_2_) = (π, π) at *θ*_3_ = 0. In contrast, the output phase was *θ*_4_ = π when (*θ*_1_, *θ*_2_) = (π, 0), (0, π), and (π, π) at *θ*_3_ = π. The Ψ-shaped interferometer functioned as a two input AND gate when *θ*_3_ = 0, and as a two input OR gate when *θ*_3_ = π. Accordingly, the phase of the resulting wave showed majority logic behaviour. Calculated waveforms for all input conditions were also overlaid as bold lines, which agreed very well with the experimental results. These results also indicate the possibility of cascaded connections, because the logical inputs and output were given by the phase format. In addition, a NOT gate can be realized by a waveguide with distance equivalent to half the wavelength. It is noteworthy that every logic function can be expressed by combinations of AND, OR, and NOT gates.

**Figure 2:**
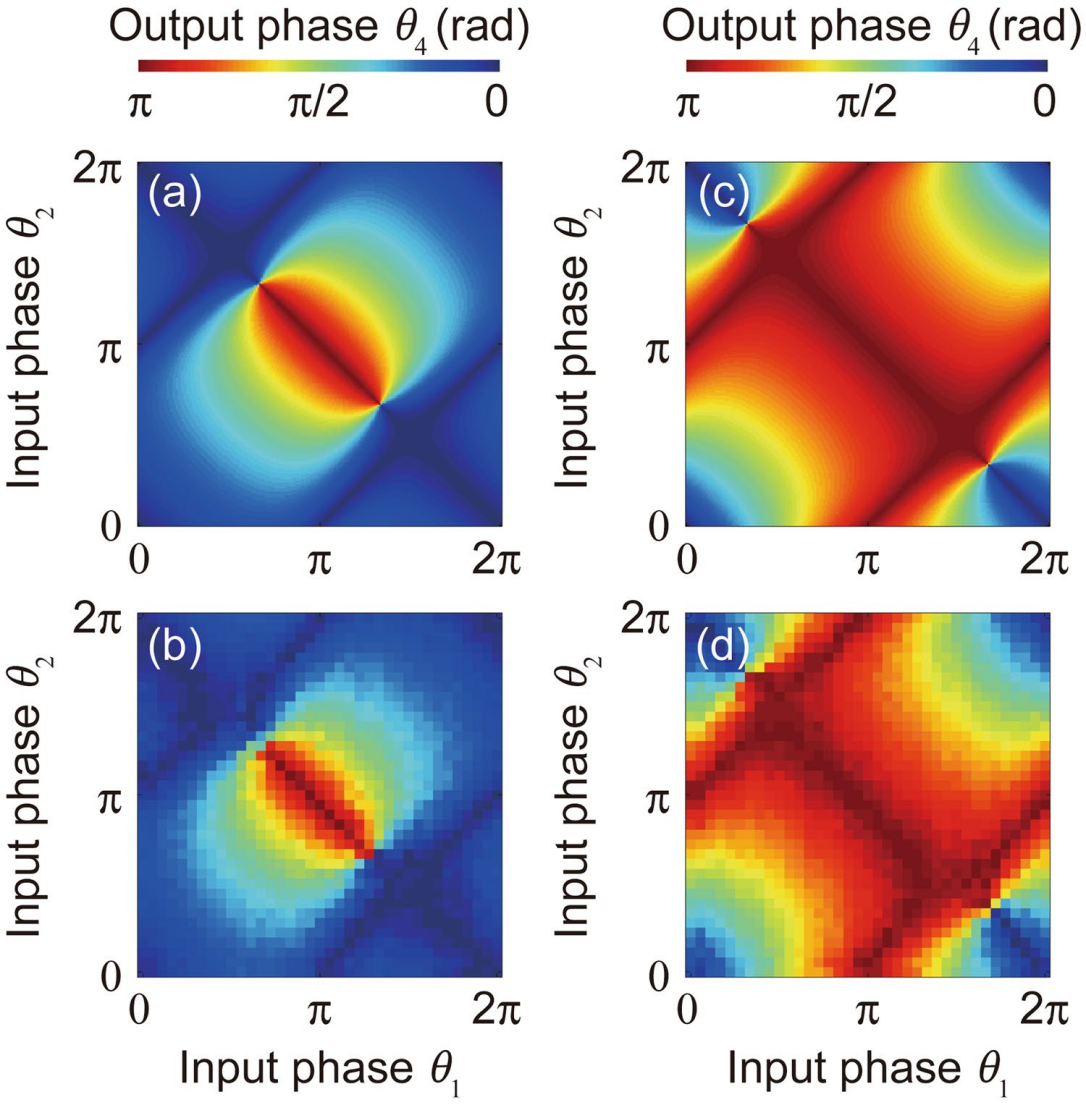
Logical output dependences for all combinations of input phases. Logical output dependence for all combination of input phases. Output phase *θ*_4_ dependence for AND-operation configuration obtained by (**a**) calculation and (**b**) experiment, and that of OR-operation configuration obtained by (**c**) calculation and (**d**) experiment. Colour represents the amount of phase shift.

Next, we scanned the output phase *θ*_4_ for all combinations of input phases. The phases of *θ*_1_ and *θ*_2_ were swept from 0° to 360° with 10° increments. The theoretical values of *θ*_4_ were calculated by Eq. (1). Calculated and experimentally obtained values of *θ*_4_ for the AND gate and OR gate are displayed in Fig. 2. Experimental results closely reproduced the calculated results, and mean deviations of *θ*_4_ in these experiments were 7.6° and 5.7° for the AND gate and OR gate, respectively. However, large deviations of *θ*_4_ were observed for specific combinations of (*θ*_1_, *θ*_2_). In Fig. 2(b), large deviations appeared in the vicinity of (*θ*_1_, *θ*_2_) = (4/3π, 2/3π) and (2/3π, 4/3π). In Fig. 2(d), large deviations appeared in the vicinity of (*θ*_1_, *θ*_2_) = (5/3π, 1/3π) and (1/3π, 5/3π). At these points, output waves were suppressed by the destructive interference of three waves, and *θ*_4_ cannot be defined. Except for these points, the observed *θ*_4_ agreed with the calculation for any combination within (*θ*_1_, *θ*_2_). Because the output phase exhibited an intermediate value between 0 and π for non-binary input phases, the experimental results indicate the potential of multivalued logic based on spin wave interference.

## Discussion

**Figure 3:**
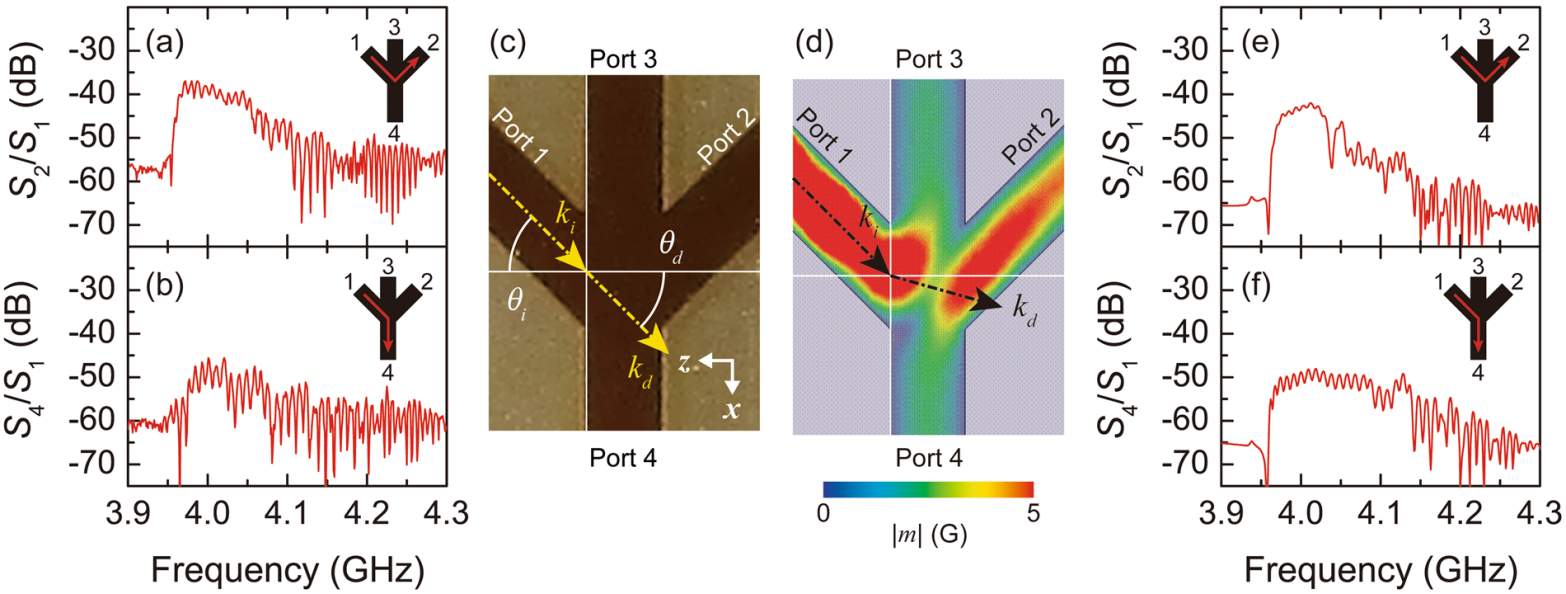
Spin wave transmission thorough the input junction. Measured transmission properties of (**a**) *S*_2_/*S*_1_ and (**b**) *S*_4_/*S*_1_. Inset pictures illustrate the flow of spin waves within the waveguide. (**c**) Schematization of spin wave diffraction at the junction area. (**d**) Simulated flow of the spin wave injected from port 1 with the homogeneous demagnetizing field *H*_*d*_ = 1707 Oe with applied field *H*_0_ = 3095 Oe. Wavenumbers *k*_*i*_ and *k*_*d*_ are overlaid with *θ*_*i*_ = 45°, *θ*_*d*_ = 15.7°. Simulated transmission properties of (**e**) *S*_2_/*S*_1_ and (**f**) *S*_4_/*S*_1_. Inset pictures illustrate the flow of spin waves within the waveguide.

The magnonic majority gate was experimentally shown using forward volume spin waves, but the propagation of spin waves in the input junction was not controlled exactly. For example, Fig. 3(a) and (b) show transmission spectra of *S*_2_/*S*_1_ and *S*_4_/*S*_1_, in which spin waves injected from port 1 were measured at port 2 and 4, respectively. In the result, the intensity of *S*_2_/*S*_1_ was clearly greater than that of *S*_4_/*S*_1_. It should be emphasised that *S*_4_/*S*_1_ represents the output signal, while *S*_2_/*S*_1_ represents a backflow of information towards another input port. The latter transmission should be prohibited, especially considering its effect on the concatenation of densely packed devices. Accordingly, the dynamics of spin waves in bent paths must be carefully investigated. Snell’s law can explain the diffraction of spin waves that are obliquely incident on the input junction^29^.

Figure 3(c) illustrates the model of spin wave injection from port 1 with incident wavenumber *k*_*i*_ and diffracted wavenumber *k*_*d*_ in the vicinity of the input junction. The resulting diffraction angle *θ*_*d*_ can be estimated by Snell’s law:

2 $${\theta }_{d}={\sin }^{-1}\frac{{k}_{i}}{{k}_{d}}\,\sin \,{\theta }_{i}.$$

Clearly, higher *θ*_*d*_ increases the intensity of *S*_4_/*S*_1_ and decreases the intensity of *S*_2_/*S*_1_. According to Eq. (2), the value of *θ*_*d*_ can be controlled by two parameters: *θ*_*i*_ and *k*_*i*_/*k*_*d*_. Because *θ*_*d*_ is proportional to *θ*_*i*_, the choice of a higher *θ*_*i*_ is quite reasonable to obtain a higher *θ*_*d*_, but the control of parameter *k*_*i*_/*k*_*d*_ is equally crucial. Figure 3(d) shows the simulated behaviour of the forward volume spin wave injected from port 1 with *θ*_*i*_ = 45°. If the value of parameter *k*_*i*_/*k*_*d*_ = 1, *θ*_*d*_ predicted from Eq. (2) becomes 45°, yielding propagation equally towards port 2 and 4. However, in the simulated result, the intensity on the port 2 side was remarkably higher than that on the port 4 side, and thus, lowering of *θ*_*d*_ was caused by reduction in the parameter *k*_*i*_/*k*_*d*_. In the ridge waveguide, wavenumber becomes a function of the waveguide width due to the lateral confinement of propagating waves, and the 350 μm-wide input waveguides gave *k*_*i*_ = 3.7 × 10^3^ m^−1^. On the other hand, the waveguide was extended in the *x*-axis direction in the input junction, and such broadening of the apparent width gave *k*_*d*_ = 9.7 × 10^3^ m^−1^. This extension of *k*_*d*_ resulted in the value of *k*_*i*_/*k*_*d*_ = 0.38, which gave the significant reduction in *θ*_*d*_ = 15.7°. Simulated transmission spectra are also displayed in Fig. 3(e) and (f). As shown in the experiment, the intensity of *S*_2_/*S*_1_ was clearly greater than that of *S*_4_/*S*_1_. Accordingly, the parameter *k*_*i*_/*k*_*d*_ can be a decisive factor in the dynamics of spin waves in the input junction. Thus Snell’s law can qualitatively explain the behaviour of spin wave propagation in the junction, but quantitative differences exist due to the modification of Snell’s law from its original form defined for plane waves propagating in a finite space (not waveguide modes).

**Figure 4:**
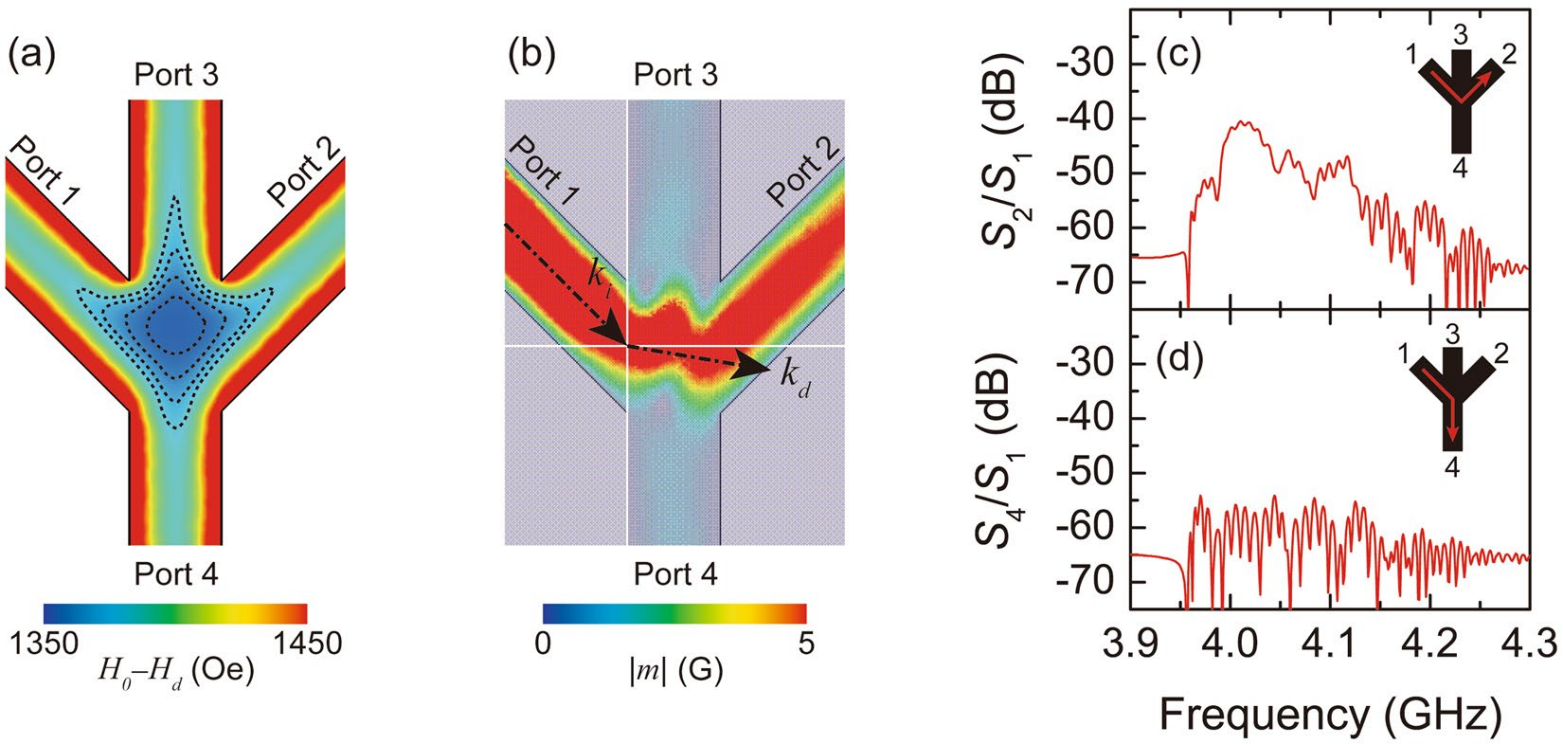
Contribution of demagnetizing field. (**a**) Simulated internal magnetic field with applied field *H*_0_ = 3095 Oe and saturation magnetization 4π*M*_*S*_ = 1759 G. A contour plot of internal magnetic field within the junction area is overlaid with dotted lines. From the inner region, dotted lines represent internal fields of 1364 Oe, 1370 Oe, 1376 Oe, 1382 Oe, and 1388 Oe. (**b**) Simulated flow of the spin wave injected from port 1 with inhomogeneous demagnetizing field with *H*_0_ = 3095 Oe. Wavenumbers *k*_*i*_ and *k*_*d*_ are overlaid with *θ*_*i*_ = 45°, *θ*_*d*_ = 9.6°. Simulated transmission properties of (**c**) *S*_2_/*S*_1_ and (**d**) *S*_4_/*S*_1_. Inset pictures illustrate the flow of spin waves within the waveguide.

Furthermore, to consider more realistic behaviour, the spatial distribution of the demagnetizing field *H*_*d*_ should be considered. Figure 4(a) displays the simulated internal magnetic field distribution in the vicinity of the input junction. It is clear that the demagnetizing field increased at the centre of the junction area due to the magnetic shape anisotropy. The minimum value in the junction area was *H*_0_−*H*_*d*_ = 1364 Oe, which was smaller than the value of 1388 Oe at the middle of the input branch. The distribution of spin waves was simulated while considering the effect of demagnetizing field, and the result is shown in Fig. 4(b). In this situation, the spin wave propagation towards port 4 was further suppressed, and the injected spin wave was steered towards port 2. This demagnetizing effect gave an extra enhancement of *k*_*d*_ = 1.59 × 10^4^ m^−1^ at the centre of the input junction, which resulted in *k*_*i*_/*k*_*d*_ = 0.23 and *θ*_*d*_ = 9.6°. Simulated transmission spectra are shown in Fig. 4(c) and (d). By considering the effect of magnetic shape anisotropy, the intensity of *S*_2_/*S*_1_ was slightly increased from −43.3 dB to −41.8 dB, while that of *S*_4_/*S*_1_ was decreased from −48.8 dB to −58.1 dB.

**Figure 5:**
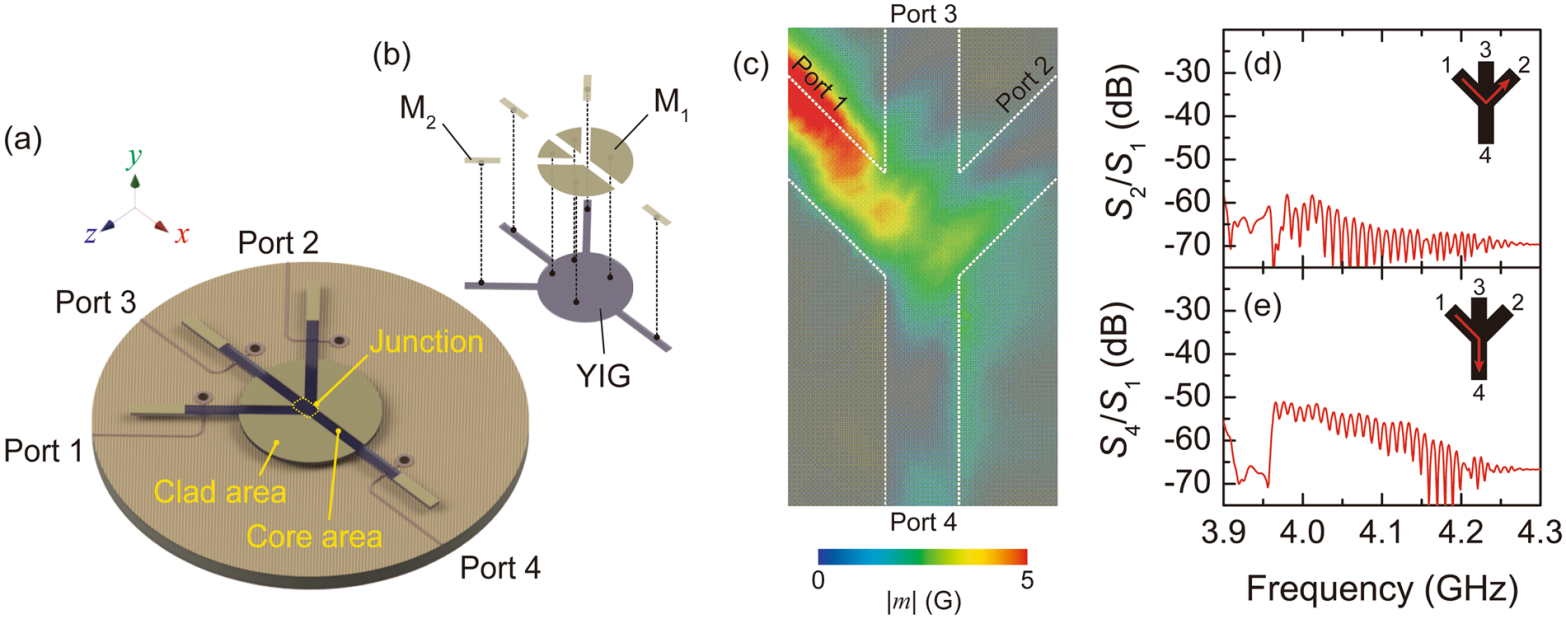
Ψ-shaped interferometer composed of core-cladding waveguide. (**a**) Schematic illustration of the interferometer. The centre of the interferometer is composed of disk-shaped YIG comprised of core-cladding waveguide. (**b**) Detail of waveguide structure. M_1_ represents 100-nm-thick gold forming a clad area. M_2_ represents 10-nm-thick gold to attenuate backscattered waves. All gold films cover a single surface of YIG film, and the uncovered side is facing the microstrip lines. (**c**) Simulated flow of the spin wave injected from port 1 with *H*_0_ = 3095 Oe. Simulated transmission properties of (**d**) *S*_2_/*S*_1_ and (**e**) *S*_4_/*S*_1_. Inset pictures illustrate the flow of spin waves within the waveguide.

To reduce the unfavourable artefact induced by demagnetization effects, we propose a new waveguide geometry formed in an un-patterned plane of YIG film, in which the magnetic shape anisotropy is not induced within the input junction. Recently, a similar structure was used by Balinskiy *et al*. for a different application^30^. Figure 5(a) and (b) illustrate schematic diagrams of majority gates, in which the input junction was composed of core-cladding waveguide (See Methods). The clad area was composed of YIG film covered by 100-nm-thick gold, in order to have sufficient change in the wavenumber from the core area^27, 31^. It is noteworthy that the effect of the lateral confinement is not strong compared with ridge-type waveguide, because the magnetization at the interface between the bare-surface waveguide and metallized waveguide is no longer the same as that at the edge of the ridge waveguide, and thus, standing waves in the lateral direction are not formed. Therefore, reduction of the parameters *k*_*i*_/*k*_*d*_ and *θ*_*d*_ is suppressed.

The proposed structure was tested by simulation. The behaviour of spin waves injected from port 1 is displayed in Fig. 5(c). Comparing to the ridge waveguide shown in Fig. 4(b), propagation towards port 4 was improved even though leakage of the propagating wave from the core area was confirmed. It should be noted that the propagation towards port 2 was clearly suppressed. Transmission spectra are also graphed in Fig. 5(d) and (e). As a result, the intensity of *S*_2_/*S*_1_ was significantly reduced, and propagation towards port 4 was rather dominant. Thus, in the core-cladding waveguide, spin wave propagation in the input junction was successfully controlled according to the predictions of Snell’s law.

In the core-cladding waveguide, the increase of the intensity *S*_4_/*S*_1_ was rather low, considering the remarkable decrease in *S*_2_/*S*_1_, because of the lack of a stop band within the clad area. This resulted in the radiation of spin waves from the core area, which is clearly visible in Fig. 5(c), especially in the vicinity of the input junction. The use of artificial lattices, such as magnonic crystals, may suppress this radiation thanks to their complete band gaps^32^. Because spin wave propagation is completely prohibited within the stop band, a sequence of defects can steer the wave flow without radiation, such as in line-defect waveguides^33, 34^. The demonstration of a majority gate composed of such waveguides is outside the scope of this work.

In conclusion, we experimentally demonstrated a magnonic majority gate using forward volume spin waves, and the dynamics of spin waves in the input junction of Ψ-shaped interferometer was investigated according to Snell’s law. Spin wave diffraction in the input junction can be controlled by the incident angle and the ratio of incident and diffracted wavenumber. It is noteworthy that the incident angle limitations cause a restriction of possible network geometries, and it would instead be preferable to optimize the wavenumber ratio. In the case of conventional ridge waveguides, lateral confinement of waves and magnetic shape anisotropy reduced the wavenumber ratio, resulting in the increase of backflow of information towards another input port. Such disadvantages may be avoided by using core-cladding waveguide. The use of forward volume spin waves enables isotropic wave flow in the device plane, and thus, more complex waveguide geometries and accompanying rich functionalities could be used. The information transmission and processing are performed simultaneously, and thus, the bus and processing unit could be integrated, which may improve the processing capability of logic circuits. The size of the presented majority gate is still larger than that of CMOS devices, owing to the use of micrometre thick YIG for this proof of principle. However, these devices are capable of further miniaturization and may be made from thinner ferromagnetic films. Thus, the results of the magnonic majority gate shown here are important for future integrated magnonic logic circuits.

## Methods

### Experimental setup

All input ports were connected to the same excitation source via the splitter, phase shifters, and attenuators to adjust the injection phase and amplitude independently. The output signal was amplified by 30 dB, and measured by an oscilloscope in real time. Binary information was encoded into the phase of input waves by using phase shifters. All input voltages were independently adjusted by microwave attenuators, so that the output peak-to-peak voltage provided from each input port was 10.2 ± 0.5 mV.

### Simulations

All electromagnetic simulations were performed using ANSYS HFSS ver. 16.1 based on a finite element method^27^. In all simulations, the saturation magnetization of YIG was defined as 1749 G. Gilbert damping of *α* = 10^−4^ was assigned for the intrinsic damping of the waveguide, and the extrinsic damping was Δ*H*_0_ = 1.9 Oe. The latter damping value was obtained by fitting the transmission intensity, which may represent extra propagation loss caused by damage to the sidewall of the ridge waveguide during the micro-sandblasting process. To investigate the distribution of internal magnetic field shown in Fig. 4(a), magnetostatic analysis was performed using COMSOL Multiphysics ver. 4.4. In this simulation, external magnetic field *H*_0_ = 3095 Oe was applied to a 10-μm-thick YIG waveguide with coercivity of 0.31 Oe and residual magnetization of 70 G. The calculated demagnetizing field distribution was divided into subdomains following the contour plot shown in Fig. 4(a), and imported into the electromagnetic simulations shown in Fig. 4(b)–(e).

### Spin wave attenuation

In the experiment, thin-gold attenuators with a length *L*_Au_ = 1.5 mm were applied to the ends of the waveguide. Spin waves entered these attenuators from the bare surface waveguide with wavenumber *k*_0_, and propagated within the attenuators with wavenumber *k*_1_. Because metallization of the waveguide surface changed the propagating wavenumber from *k*_0_ to *k*_1_, spin waves were slightly reflected when they entered the attenuator. Under the magnetostatic approximation, the amplitude of reflection can be written by (*k*_0_ − *k*_1_)/(*k*_0_ + *k*_1_), and that of transmission can be written by 2*k*_0_/(*k*_0_ + *k*_1_) in the case of vertical incidence, to support continuity of scalar potentials at the boundary. After spin waves entered the attenuator, they propagated towards the end of the waveguide while attenuated, and backscattered towards the entrance with a total propagation distance of 2*L*_Au_. The decay envelope of the propagating wave at position *x* is proportional to exp(−*x*/*L*_att_), where *L*_att_ is the attenuation length. Thus, the reflection amplitude from the waveguide end can be written as exp(−2*L*_Au_/*L*_att_), and the reflected wave leaked from the attenuators has a transmission coefficient 2*k*_1_/(*k*_0_ + *k*_1_). Thus, total reflection from the thin-gold attenuator can be given by

3 $$r=\frac{1}{{k}_{0}+{k}_{1}}\{{k}_{0}-{k}_{1}+4\frac{{k}_{0}{k}_{1}}{{k}_{0}+{k}_{1}}{e}^{-2{L}_{{\rm{Au}}}/{L}_{{\rm{att}}}}\}.$$

Values of *k*_0_ = 7.2 × 10^3^ m^−1^, *k*_1_ = 6.3 × 10^3^ m^−1^ and *L*_att_ = 0.62 mm were obtained by the electromagnetic simulation when gold thickness was 10 nm^27^. Using these obtained values, *r* = 7.2% was estimated. In the realistic case, the value of *r* may be further reduced due to the lossy scattering at the waveguide end. The value of *k*_0_ obtained by the electromagnetic simulation was larger than 3.7 × 10^3^ m^−1^ given by the exact solution with the waveguide width of 350 μm. This may be explained by the Goos-Hänchen effect at the waveguide edge^35^.

### Core-cladding waveguide

Similar to the optical fibres, a core area propagating waves with large wavenumber *k*_core_ was surrounded by a clad area propagating waves with small wavenumber *k*_clad_. Because the wavenumber of spin waves in the bare-surface waveguide is approximately double of that in the metallized waveguide when the metal layer is sufficiently thick^27, 36^, the ratio of *k*_clad_/*k*_core_ = 1/2 was obtained. Thus, when spin waves enter from the bare-surface side to the metallized side, the diffraction angle *θ*_*d*_ = 90° is given by Eq. (2) with the critical incident angle of *θ*_*c*_ = 30°, and the core-cladding waveguide is capable of steering waves satisfying *θ*_*i*_ > *θ*_*c*_. It is noteworthy that this core-cladding waveguide never induces magnetic shape anisotropy.
